# Treatment with anti-IL-6 receptor antibody prevented increase in serum hepcidin levels and improved anemia in mice inoculated with IL-6–producing lung carcinoma cells

**DOI:** 10.1186/s12885-016-2305-2

**Published:** 2016-04-11

**Authors:** Mariko Noguchi-Sasaki, Yusuke Sasaki, Yasushi Shimonaka, Kazushige Mori, Kaori Fujimoto-Ouchi

**Affiliations:** Product Research Department, Chugai Pharmaceutical Co., Ltd., 200 Kajiwara, Kamakura, Kanagawa 247-8530 Japan

**Keywords:** Interleukin-6, Anemia, Hepcidin, Cancer, Iron metabolism

## Abstract

**Background:**

Hepcidin, a key regulator of iron metabolism, is produced mainly by interleukin-6 (IL-6) during inflammation. A mechanism linking cancer-related anemia and IL-6 through hepcidin production is suggested. To clarify the hypothesis that overproduction of IL-6 elevates hepcidin levels and contributes to the development of cancer-related anemia, we evaluated anti-IL-6 receptor antibody treatment of cancer-related anemia in an IL-6–producing human lung cancer xenograft model.

**Methods:**

Nude mice were subcutaneously inoculated with cells of the IL-6–producing human lung cancer cell line LC-06-JCK and assessed as a model of cancer-related anemia. Mice bearing LC-06-JCK were administered rat anti-mouse IL-6 receptor antibody MR16-1 and their serum hepcidin levels and hematological parameters were determined.

**Results:**

LC-06-JCK–bearing mice developed anemia according to the production of human IL-6 from xenografts, with decreased values of hemoglobin, hematocrit, and mean corpuscular volume (MCV) compared to non–tumor-bearing (NTB) mice. LC-06-JCK–bearing mice showed decreased body weight and serum albumin with increased serum amyloid A. MR16-1 treatment showed significant inhibition of decreased body weight and serum albumin levels, and suppressed serum amyloid A level. There was no difference in tumor volume between MR16-1-treated mice and immunoglobulin G (IgG)-treated control mice. Decreased hemoglobin, hematocrit, and MCV in LC-06-JCK–bearing mice was significantly relieved by MR16-1 treatment. LC-06-JCK–bearing mice showed high red blood cell counts and erythropoietin levels as compared to NTB mice, whereas MR16-1 treatment did not affect their levels. Serum hepcidin and ferritin levels were statistically elevated in mice bearing LC-06-JCK. LC-06-JCK–bearing mice showed lower values of MCV, mean corpuscular hemoglobin (MCH), and serum iron as compared to NTB mice. Administration of MR16-1 to mice bearing LC-06-JCK significantly suppressed levels of both serum hepcidin and ferritin, with increased values of MCV and MCH.

**Conclusions:**

Our results suggest that overproduction of hepcidin by IL-6 signaling might be a major factor that leads to functionally iron-deficient cancer-related anemia in the LC-06-JCK model. We demonstrated that inhibition of the IL-6 signaling pathway by MR16-1 treatment resulted in significant recovery of iron-deficiency anemia and alleviation of cancer-related symptoms. These results indicate that IL-6 signaling might be one possible target pathway to treat cancer-related anemia disorders.

## Background

Anemia is a major source of morbidity and mortality worldwide [1]. Anemia is a common hematological abnormality in cancer patients; it impairs quality of life and is associated with poorer response to the clinical treatment and a worse prognosis. Therefore, a substantial number of cancer patients require anemia treatment [2, 3]. Low hemoglobin (Hb) levels correlate with poor performance status in cancer patients [3].

Iron is an essential element for mammals as it is a component of many key redox enzymes and oxygen storage and transporting proteins such as Hb and myoglobin [4]. Iron is strictly conserved, and iron from the Hb of senescent red blood cells is recycled to provide iron for new red blood cells. Dietary iron is absorbed predominantly in the duodenum to replace the small daily losses. Because mammals lack mechanisms to excrete excess iron, intestinal iron absorption is regulated by a feedback mechanism [5].

Hepcidin is a 25-amino acid peptide hormone produced mainly in the liver that regulates intestinal iron absorption by causing degradation of the enterocyte iron transporter ferroportin, iron recycling by macrophages, and iron release from hepatic stores [4, 6]. Hepcidin secretion is regulated by iron stores, oxygenation, and inflammatory signals.

Interleukin-6 (IL-6) plays a major role in the response to injury and is involved in the immune response, inflammation, and hematopoiesis. IL-6 exerts various biological activities on responding cell populations through its binding to transmembrane IL-6 receptor as well as to soluble IL-6 receptor. Dysregulated continuous production of IL-6 by a distinct cell population plays a pathological role in various inflammatory autoimmune diseases. Moreover, IL-6 has been suggested to be involved in the pathology of cancer. High levels of circulating IL-6 are observed in almost all types of cancer and predict a poor outcome [7]. IL-6 levels correlate negatively with Hb levels in advanced untreated epithelial ovarian cancer patients [8]. There is direct evidence that recombinant human IL-6 (rhIL-6) treatment as an antitumor immunotherapy induces anemia in cancer patients and that this anemia is reversible after the cessation of rhIL-6 treatment [9].

IL-6 is a key factor in inducing hepcidin production. IL-6 directly regulates hepcidin through induction and subsequent promoter binding of signal transducer and activator of transcription 3 (STAT3) [10]. IL-6 has been reported to elevate hepcidin mRNA expression levels in freshly isolated mouse hepatocytes and in cells of the human hepatoma cell line HepG2 [11, 12]. Moreover, there is a report that immunodeficient mice bearing hepcidin-producing tumor xenografts developed severe anemia despite abundant dietary iron, and had lower serum iron and increased hepatic iron compared with mice with control tumors [13]. In a clinical report, injection of rhIL-6 into healthy human volunteers led to an increase in urinary hepcidin-25 levels and a decrease in serum iron [14]. Tocilizumab, a humanized anti-IL-6 receptor antibody, rapidly down-regulated circulating hepcidin levels in two cases of multicentric Castleman’s disease (MCD) [15]. Tocilizumab improved anemia of inflammation in MCD accompanied by down-regulation of hepcidin, suggesting that IL-6 plays an essential role in the induction of hepcidin in MCD, although multiple factors can affect serum hepcidin levels [16]. These reports imply a relationship between IL-6 and hepcidin production in some disorders.

In a previous study, we established a model of cancer-related anemia in mice by the subcutaneous inoculation of cells of the IL-6–producing human lung cancer cell line LC-06-JCK [17]. The model showed elevated levels of IL-6 in serum. Hb levels significantly decreased in the model compared with non–tumor-bearing (NTB) mice, and the decreased Hb levels were reversed by treatment with the rat anti-mouse IL-6 receptor antibody MR16-1. Although MR16-1 is expected to have a strong effect on increasing Hb levels, the effects of MR16-1 on cancer-related anemia in terms of iron metabolism are not fully understood. Therefore, in the present study, we investigated whether treatment with MR16-1 affects iron metabolism, and we evaluated the contribution of hepcidin in cancer-related anemia in the LC-06-JCK mouse model.

## Methods

### Cancer cells

The human cancer cell line LC-06-JCK (lung clear cell carcinoma) was obtained from the Central Institute for Experimental Animals (Kanagawa, Japan) and was maintained in vivo in male CAnN.Cg-Foxn1^nu^/CrlCrlj nu/nu mice (nude mice; Charles River Laboratories Japan, Kanagawa, Japan) as previously reported [18].

### Animal models

Five-week-old male nude mice were obtained from Charles River Laboratories Japan. All animals were maintained under specific pathogen free conditions and allowed to acclimatize and recover from shipping-related stress for at least 1 week in our animal facility before use. The health of the mice was monitored by daily observation.

Tumors of LC-06-JCK grown in donor nude mice were resected, and small pieces were subcutaneously inoculated into host nude mice, as previously described [18]. Mice were fed irradiated rodent chow and chlorinated water *ad libitum*. The animals were kept in a controlled light–dark cycle (12–12 h). Animal procedures were approved by the Institutional Animal Care and Use Committee at Chugai Pharmaceutical Co., Ltd. All animal experiments were performed in accordance with the Guidelines for the Care and Use of Laboratory Animals at Chugai Pharmaceutical Co., Ltd.

### Administration of rat anti-mouse IL-6 receptor monoclonal antibody

Rat anti-mouse IL-6 receptor antibody MR16-1 was produced by the method previously reported [19]. In the first experiment, the mice were randomly allocated to control and treatment groups. MR16-1 was administered intraperitoneally at a dose of 20 mg/kg once a week to male nude mice bearing LC-06-JCK tumors, starting 15 days after inoculation with LC-06-JCK tumor pieces, when the tumors were sufficiently established in the mice. Mice in the control group were administered an equal dose of rat immunoglobulin G (IgG) purchased from MP Biomedicals (Solon, Ohio, USA). Rat IgG was dissolved in distilled water (Otsuka Pharmaceutical, Tokyo, Japan) and both MR16-1 and rat IgG were diluted to appropriate concentrations with saline (Otsuka Pharmaceutical). Mice were euthanized by exsanguination under anesthesia with isoflurane before starting treatment with MR16-1 or rat IgG (0 weeks), or at 2 or 4 weeks after start of treatment.

In the second experiment, the mice were randomly allocated to control and treatment groups. MR16-1 was intraperitoneally administered at a dose of 40 mg/kg once a week to male nude mice bearing LC-06-JCK tumors, starting 16 days after inoculation with LC-06-JCK tumor pieces. Mice were euthanized by exsanguination under anesthesia with isoflurane, and blood was collected before starting treatment with MR16-1 or rat IgG (0 weeks) or 5 weeks (1 week after final administration of MR16-1 or rat IgG) after start of treatment. The tumor volume was estimated by using the equation V = *ab*^2^/2, where *a* and *b* are tumor length and width, respectively. Tumor volume and body weights were measured in the morning.

### Specimen collection

Mice were euthanized by exsanguination under anesthesia with isoflurane, and blood was collected into Minicollect ethylenediaminetetraacetic acid (EDTA) tubes and Minicollect serum tube (Greiner Bio-One, Kremsmünster, Austria). Blood samples were analyzed immediately to determine hematological parameters, and serum was isolated according to the manufacturer’s instructions and stored at −80 °C until use for assays.

### Measurement of hematological and iron-related parameters and cytokines

Hematological parameters were measured by an automated hematology analyzer KX-21NV (Sysmex Corporation, Hyogo, Japan). The levels of cytokines and albumin present in serum were determined by using commercially available ELISA kits for human IL-6, mouse erythropoietin (EPO) (R&D Systems, Minneapolis, MN, USA), mouse serum amyloid A (Life Technologies Japan, Tokyo, Japan), mouse albumin (Shibayagi, Gunma, Japan), and ferritin (ALPCO Diagnostics, Salem, NH, USA). Serum iron level was determined by QuantiChrom Iron Assay Kit (BioAssay Systems, Hayward, CA, USA). Mouse interleukin-1β (IL-1β), tumor necrosis factor-α (TNF-α), and IL-6 were measured by Bio-Plex Pro cytokine assays according to the manufacturer’s instructions (Bio-Rad Laboratories, Hercules, CA, USA). The assays were performed using the Bio-Plex Pro II wash station with magnetic plate carrier, and cytokines were determined by the Bio-Plex 200 System (Bio-Rad Laboratories).

### Measurement of mouse serum hepcidin-25

Concentrations of mouse serum hepcidin were measured by a sensitive liquid chromatography/electrospray ionization tandem mass spectrometry (LC/ESI–MS/MS) method using a 4000 QTRAP (AB Sciex, Foster City, CA, USA) equipped with an ACQUITY Ultra Performance LC system (Waters, Tokyo, Japan) as previously reported [20, 21].

### Statistical analysis

Statistical analysis was performed by Wilcoxon test using JMP software (SAS Institute, Cary, NC, USA). A *P* value of <0.05 was considered statistically significant. Data are represented as mean and SD.

## Results

### LC-06-JCK–bearing mice developed anemia with decreased values of Hb, hematocrit, and MCV with the elevation of human IL-6 levels produced from xenografts

To further investigate the anemia observed in the LC-06-JCK–bearing mice reported in our previous study, we first confirmed the reproducibility of our established experimental model in terms of development of anemia and production of human IL-6 from the xenograft.

**Fig. 1:**
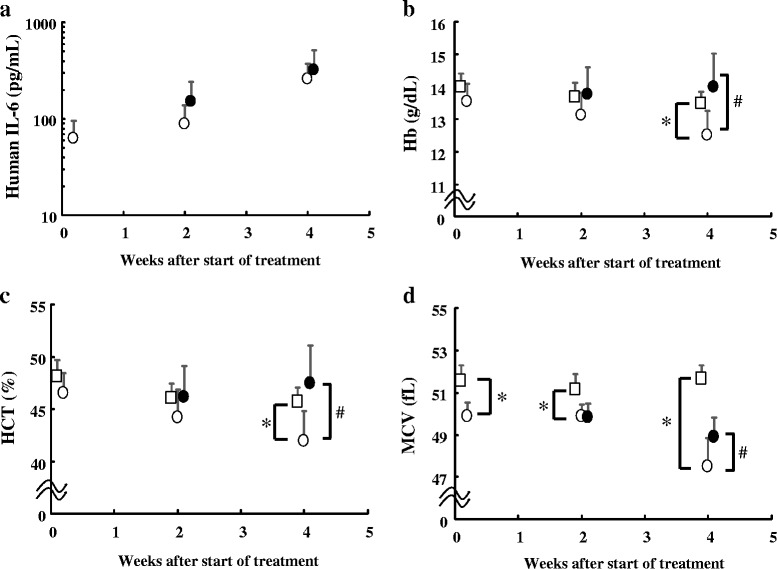
Changes in the parameters during the development of anemia in the LC-06-JCK mouse model. **a** Human IL-6 levels, **b** Hb levels, **c** HCT levels, and **d** MCV values were measured in mice treated for 0, 2, and 4 weeks. Open squares, NTB group; open circles, TB group; closed circles, MR16-1 group. Results are the mean + SD for 8 mice in each group. * *P* < 0.05, age-matched NTB group versus TB group (Wilcoxon test); # *P* < 0.05, TB group versus MR16-1 group (Wilcoxon test)

We detected high levels of human IL-6 in mice in the IgG-treated LC-06-JCK–bearing control group (TB group) in a time-dependent manner, and we confirmed that IL-6 was produced in levels as high as previously reported [17] (Fig. 1a). We also confirmed that we were not able to detect human IL-6 in mice in the NTB group as they did not bear tumors. The values of Hb, hematocrit (HCT), and mean corpuscular volume (MCV) were lower in the TB group than the respective values in the NTB group at 4 weeks (Fig. 1b–d). We observed no significant differences in human IL-6 levels between LC-06-JCK–bearing mice with or without MR16-1 treatment. MR16-1 treatment significantly reversed the decline of Hb, HCT, and MCV values in this model.

### MR16-1 treatment ameliorated cancer-related symptoms in the LC-06-JCK model

Diseases associated with overexpression of hepcidin are often microcytic and hypochromic [22]. Because the anemia in this model was observed to be microcytic, we used the model to investigate iron-related parameters in order to clarify the hypothesis that overproduction of IL-6, a positive regulator of hepcidin, elevates hepcidin levels and disturbs iron metabolism.

We first investigated cancer-related symptoms in these mice. Body weight loss was observed in the TB group as compared to the NTB group at 5 weeks after start of treatment, and MR16-1-treated LC-06-JCK–bearing mice (MR16-1 group) showed a significant increase in body weight as compared to the TB group (Table 1). We confirmed that, as previously reported, albumin levels decreased in the TB group and that levels of albumin increased after 5-week treatment with MR16-1.

**Table 1:** Effects of MR16-1 on cancer-related symptoms in the LC-06-JCK mouse model

	0 weeks		5 weeks		
	NTB	TB	NTB	TB	MR16-1
Body weight (g)	25.5 ± 1.4	25.8 ± 1.3	28.0 ± 1.1	24.5 ± 2.0^*^	25.9 ± 2.0^**^
Albumin (mg/mL)	28.9 ± 3.0	26.5 ± 5.1^*^	25.5 ± 3.8	16.7 ± 2.6^*^	22.1 ± 2.8^**^
SAA (μg/mL)	60.7 ± 95.7	589.2 ± 614.8^*^	24.8 ± 12.7	5638.0 ± 2867.5^*^	353.0 ± 302.9^**^
Tumor volume (mm^3^)	ND	143.9 ± 43.0	ND	1369.9 ± 301.5	1647.6 ± 515.8

Body weight, serum albumin levels, SAA levels, and tumor volumes were measured in mice before treatment (0 weeks) and after being treated for 5 weeks. Results are the mean ± SD for 19–20 mice in each group. *P < 0.05, age-matched NTB group versus TB group (Wilcoxon test); **P < 0.05, TB group versus MR16-1 group (Wilcoxon test)

IL-6 is known to be involved in the inflammatory response; thus, we examined whether the acute-phase reaction was elevated in this model. We measured the levels of serum amyloid A (SAA), a hepatic acute-phase reactant that is known to be increased in response to inflammation and certain cytokines, particularly IL-6 [23]. The TB group had a dramatic increase in circulating SAA levels compared to the NTB group at 0 weeks and after treatment for 5 weeks. On the other hand, the elevation of SAA levels was significantly inhibited in the MR16-1 group as compared to the TB group. We also assessed whether inhibition of the IL-6 signaling pathway affected tumor volume. There were no differences in tumor volume between the TB and MR16-1 groups (Table 1).

### Treatment with MR16-1 relieved decreased Hb and HCT despite lack of change in red blood cell counts and erythropoietin levels in the LC-06-JCK model

**Fig. 2:**
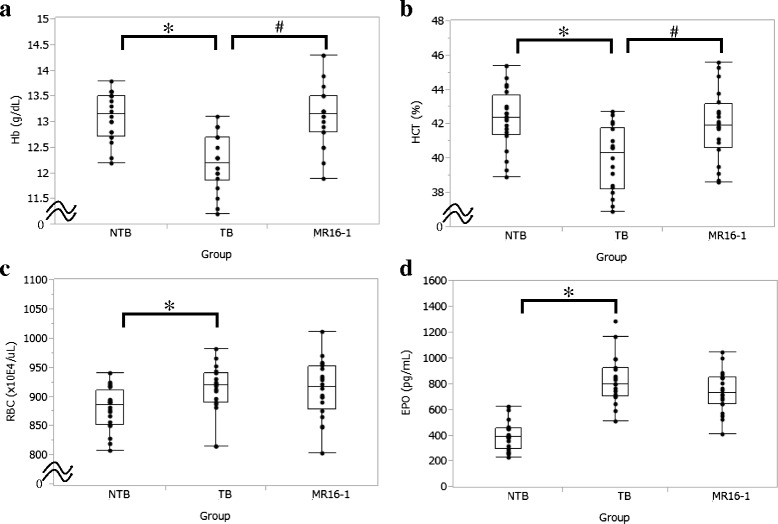
Effects of MR16-1 on hematological parameters in the LC-06-JCK mouse model. **a** Hb levels, **b** HCT levels, **c** RBC counts, and **d** serum EPO levels were measured in mice treated for 5 weeks. Results are from 18–20 mice in each group. * *P* < 0.05, age-matched NTB group versus TB group (Wilcoxon test); # *P* < 0.05, TB group versus MR16-1 group (Wilcoxon test)

We next measured hematological parameters to assess the status of anemia in this model. Hb and HCT levels declined in the TB group as compared to the NTB group, and the decrease in each of these parameters was significantly inhibited by MR16-1 treatment (Fig. 2a–b). Red blood cell (RBC) counts were elevated in the TB group as compared to the NTB group, and treatment with MR16-1 did not affect RBC counts (Fig. 2c). EPO levels were elevated in the TB group as compared to the NTB group; however, we were not able to detect a significant difference between EPO levels in the TB and MR16-1 groups (Fig. 2d).

### Treatment with MR16-1 inhibited the increase in hepcidin and ferritin levels in the LC-06-JCK model

**Fig. 3:**
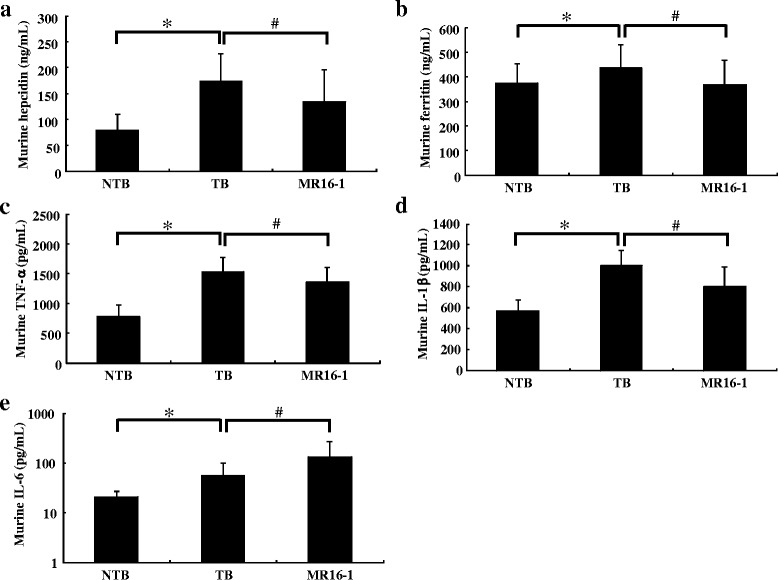
Effects of MR16-1 on iron indices and pro-inflammatory cytokines in the LC-06-JCK mouse model. **a** Murine hepcidin levels, **b** murine ferritin levels, **c** murine TNF-α levels, **d** murine IL-1β levels, and **e** murine IL-6 levels were measured in mice treated for 5 weeks. Results are the mean + SD for 19–20 mice in each group. * *P* < 0.05, age-matched NTB group versus TB group (Wilcoxon test); # *P* < 0.05, TB group versus MR16-1 group (Wilcoxon test)

We hypothesized that functional iron deficiency—which is often observed in anemia of inflammation—might be occurring in LC-06-JCK–bearing mice, and we investigated iron metabolism-related parameters to assess anemia status. Since IL-6 is a key inflammatory regulator of hepcidin, which is the major regulator of iron metabolism, we examined whether circulating hepcidin levels were elevated in LC-06-JCK–bearing mice. The TB group showed high levels of serum hepcidin at 5 weeks after treatment as compared to NTB mice (Fig. 3a). Moreover, the administration of MR16-1 statistically decreased the elevation of circulating hepcidin levels in LC-06-JCK–bearing mice.

As the iron-regulatory hormone hepcidin was increased in this model, we next measured serum ferritin levels, which correlate with iron stores in most physiological and pathological conditions. The TB group showed higher levels of ferritin as compared to the NTB group; moreover, MR16-1 treatment reduced ferritin levels in LC-06-JCK–bearing mice (Fig. 3b). As it is known that ferritin synthesis is driven by inflammation, we measured pro-inflammatory cytokines that are reported to affect ferritin levels to explore the contribution of these cytokines to ferritin levels. TNF-α and IL-1β levels were elevated in the TB group as compared to the NTB group, and treatment with MR16-1 decreased these levels (Fig. 3c–d). In contrast, although mouse IL-6 levels were also elevated in the TB group as compared to the NTB group, MR16-1 treatment led to a further increase in IL-6 levels (Fig. 3e).

### Treatment with MR16-1 inhibited the decrease in MCV and MCH values, leading to improvement of iron-deficiency anemia in the LC-06-JCK model

**Fig. 4:**
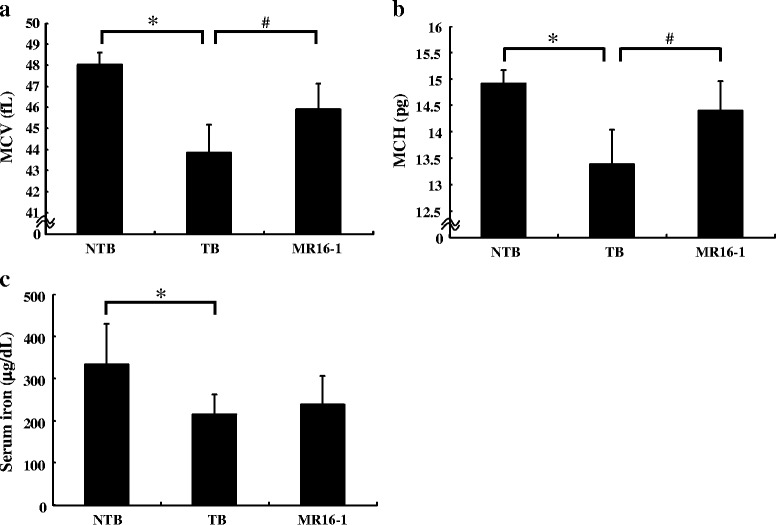
Effects of MR16-1 on iron metabolism-related parameters in the LC-06-JCK mouse model. **a** MCV values, **b** MCH values, and **c** serum iron levels were measured in mice treated for 5 weeks. Results are the mean + SD for 19–20 mice in each group. * *P* < 0.05, age-matched NTB group versus TB group (Wilcoxon test); # *P* < 0.05, TB group versus MR16-1 group (Wilcoxon test)

To investigate whether reduced iron availability was occurring in this model, we analyzed parameters of circulating erythrocytes and serum iron levels. MCV values decreased in the TB group as compared to the NTB group. On the other hand, MR16-1 treatment markedly increased MCV values in this anemia model (Fig. 4a). Mean corpuscular hemoglobin (MCH) values also decreased in the TB group as compared to the NTB group, and MR16-1 treatment increased MCH values in LC-06-JCK–bearing mice (Fig. 4b). The TB group showed statistically low levels of serum iron as compared to the NTB group (Fig. 4c). We were not able to detect any significant difference in serum iron levels between the TB and MR16-1 groups treated for 5 weeks.

## Discussion

We previously established an LC-06-JCK mouse model of anemia that showed high levels of IL-6 and low levels of Hb. In this study, we further investigated iron metabolism to assess whether the blockade of IL-6 signaling could prevent anemia in this IL-6–overproducing xenograft model.

MCV and HCT levels were observed to decrease with the development of cancer-related anemia, implying that the anemia observed in this model was microcytic (Fig. 1c–d). The TB group showed anemia as Hb and HCT levels that were significantly lower than the NTB group, with elevated RBC counts and EPO levels (Fig. 2). MR16-1 treatment inhibited the decrease of Hb and HCT levels and improved anemia in the LC-06-JCK model. Elevated EPO levels were observed in the TB group, implying that the response to anemia was not impaired in this mouse model; therefore, EPO production might increase as a compensatory response to the anemia status. MR16-1 treatment inhibited the decrease of Hb and HCT levels and improved anemia without having an impact on RBC counts or EPO levels in the LC-06-JCK model. Hypoxic and anemic conditions are known to increase the synthesis of the hormone EPO which leads to the production of erythrocytes [24]; therefore, we measured EPO levels in this mouse model. Although EPO production and RBC counts were elevated in the TB group, anemia with decreased Hb levels was observed; therefore, disturbance of iron metabolism might be the underlying cause of the anemia observed in the LC-06-JCK model. We therefore investigated iron metabolism-related parameters to assess anemia status. The TB group showed high levels of hepcidin and ferritin with low levels of MCV, low MCH values, and low serum iron (Figs. 3 and 4); the microcytic and hypochromic anemia observed in the LC-06-JCK–bearing mice suggested that increased hepcidin production might have inhibited iron utilization and led to iron-deficiency anemia. Therefore, the major cause of anemia observed in LC-06-JCK–bearing mice might be the increase in hepcidin production through IL-6 signaling leading to suppression of iron utilization. The increased MCV values, MCH values, and Hb levels after MR16-1 treatment demonstrate the effective utilization of iron for Hb synthesis (Figs. 2 and 3). Importantly, treatment of LC-06-JCK–bearing mice with MR16-1 inhibited the increase in serum hepcidin levels induced by IL-6 signaling (Fig. 3). These results imply that MR16-1 ameliorated iron-deficiency anemia via reduction of hepcidin, which consequently led to the amelioration of iron deficiency.

We hypothesized that excess hepcidin may interfere with erythropoiesis by inhibiting iron release from hepatic storage as ferritin levels were high in this model and by inhibiting iron uptake from diet and export from enterocytes to blood as serum iron level was decreased, consequently possibly causing iron deficiency in the bone marrow, leading to decreased Hb and MCV levels. Blockade of IL-6 signaling might inhibit these phenomena in this model. In anemia of chronic disease (ACD), IL-6 is reported to suppress erythropoiesis by a direct mechanism and by indirect mechanisms mediated by other cytokines. IL-6 directly inhibited proliferation of burst-forming units-erythroid (BFU-E) and colony-forming units-erythroid (CFU-E) in bone marrow cultures from healthy donors and from rheumatoid arthritis patients [25]. Elevated levels of IL-6 were found in the supernatants of bone marrow cultures from patients with ACD, suggesting that IL-6 local production in bone marrow is possibly associated with ACD [26, 27]. Moreover, IL-6 is reported to impair mitochondrial function in maturating erythroid cells resulting in impaired Hb production and erythroid maturation [28]. IL-6 seems to play several roles in the anemia of cancer by acting at the level of the bone marrow by inducing ineffective erythropoiesis and at the level of the reticuloendothelial system by changing iron diversion by up-regulating hepcidin.

Although hepcidin levels statistically decreased in the MR16-1 group as compared to the TB group, the suppression was partial (Fig. 3a). Similar phenomena was reported that hepcidin is up-regulated in multiple myeloma patients by both IL-6-dependent and -independent mechanisms [29]. Hepcidin-independent mechanisms are also reported to play an important role in murine models of anemia of cancer [30]. These results suggest that hepcidin is regulated mainly by IL-6 but that other factors may also contribute to its regulation. For example, hepcidin is known to be up-regulated by some cytokines in inflammation; on the other hand, EPO is known as a factor to decrease hepcidin expression levels [21]. There is some evidence indicating that EPO decreases hepcidin mRNA expression levels in several cell lines and mice [11, 12, 31]. However, in our results, there was no difference in serum EPO levels between IgG-treated and MR16-1–treated LC-06-JCK–bearing mice. So, although EPO production might increase as a compensatory response to the anemia status, MR16-1 treatment improved anemia without decreasing EPO levels, and EPO is reported to decrease hepcidin expression levels [21]. From all of these results taken together, and because no significant difference between EPO levels in the TB and MR16-1 groups was observed, EPO have little contribution to the reduction of hepcidin level by MR16-1 treatment. Therefore, at least, the contribution of EPO to hepcidin levels can be excluded (Fig. 2d). Shortage of serum iron was observed as the increased demand of iron for erythropoiesis, as shown in the increase of MCV levels was still partial in MR16-1 treated mice. Therefore, we think that EPO is still required to enhance erythropoiesis in LC-06-JCK mice, although MR16-1 treatment improved anemia status.

We observed the decrease of pro-inflammatory cytokines—TNF-α and IL-1β, for example—their decrease was also partial (Fig. 3). Some acute-phase proteins may still interfere with some aspects of iron metabolism despite statistically restored Hb and hepcidin levels. It is reported that α-1-antitrypsin blocks erythroid-cell iron uptake and impairs erythropoiesis [32]. TNF-α, known as an important contributor to the syndrome of cancer cachexia and other chronic inflammatory conditions, diseases in which ferritin levels are frequently altered, and another pro-inflammatory cytokine interleukin-1α (IL-1α) transcriptionally induces the H chain of ferritin, suggesting that pathways related to inflammation and stress can have an impact on ferritin regulation [33]. We observed high levels of TNF-α and IL-1β in the TB group, and MR16-1 treatment decreased these levels (Fig. 3). Ferritin levels also increased in the TB group, and these cytokines may contribute to this production. Mouse IL-6 levels increased in the MR16-1 group as compared to the TB group; on the other hand, the elevation of SAA levels, downstream of IL-6 signaling, was significantly inhibited in the MR16-1 group (Table 1). Because IL-6 is thought to be an important cause of the anemia of inflammation by inducing hepcidin expression [14], we used SAA levels as an indicator of IL-6 activity. These results suggest that IL-6 signaling was sufficiently blocked by MR16-1 treatment. Since SAA is an acute-phase reactant that is produced in the liver during acute-phase response particularly by IL-6 [23], our results suggest that IL-6 signaling is down-regulated in the liver of MR16-1-treated TB mice. Similar findings have been reported in that although tocilizumab treatment decreased TNF-α and IL-1β levels, serum IL-6 levels were increased by tocilizumab therapy regardless of the improvements in clinical measures in rheumatoid arthritis patients [34]. It is reported that levels of IL-6 in serum increase when IL-6 receptor is blocked by tocilizumab because IL-6 receptor-mediated consumption of IL-6 is inhibited by the unavailability of tocilizumab-free IL-6 receptors [35]. Alleviation of cancer-related symptoms by MR16-1 treatment seems to contribute to the reduction of TNF-α and IL-1β levels rather than the MR16-1 treatment having a direct impact on their levels by the blockade of IL-6 signaling. Importantly, although IL-6 levels, a positive regulator of hepcidin, were increased after MR16-1 treatment, hepcidin levels were significantly decreased with MR16-1 treatment (Fig. 3a); therefore, it is suggested that the improvement of iron-deficiency status might be the main mechanism of the alleviation of anemia by MR16-1 treatment. Further analysis is needed to reveal the precise mechanisms underlying cancer-related anemia.

Serum iron levels decreased in the TB group as compared to the NTB group; however, we were not able to detect any significant difference in serum iron levels resulting from MR16-1 treatment (Fig. 4c). As a possible explanation, neutralizing IL-6 signaling by MR16-1 increases erythropoiesis in bone marrow, which leads to an increase in the demand for iron to produce iron-sufficient Hb synthesis. Consequently, the release of iron into plasma from iron storage is not sufficient to maintain homeostatic iron levels. In fact, a similar transient discrepancy between bone marrow iron availability and requirements during increased erythropoietic activity is observed despite the use of oral iron supplements in chronic dialysis patients and healthy individuals who received EPO treatment for anemia [36]. EPO therapy induced a reduction in serum iron and desaturation of transferrin, which is associated with iron-deficient erythropoiesis [37]. It seems that increased erythropoietic activity induces transient serum iron deficiency.

We observed inhibition of decreased body weight and serum albumin levels, and suppressed SAA level in the LC-06-JCK model mice treated with MR16-1 (Table 1). We demonstrated that inhibition of IL-6 signaling by MR16-1 treatment resulted in not only the reduction of elevated hepcidin levels which led to the improvement of anemia status but also the systemic amelioration of disease symptoms. The role of IL-6 in cancer progression has been reported [38]. In this study, we were not able to detect any differences in tumor growth between the TB and MR16-1 groups (Table 1), and there was no correlation between tumor volume and human IL-6 levels (data not shown), suggesting that amelioration of cancer-related symptoms was not due to regression of tumors in the LC-06-JCK model. Importantly, MR16-1 interacts only with the murine IL-6 receptor and is unable to cross-react with the human IL-6 receptor [19]. On the other hand, human IL-6 binds to both human and murine IL-6 receptor [39]; therefore, human IL-6 expressed by tumor cells can act on murine IL-6 receptor expressed in mice.

The involvement of IL-6 has been emphasized in certain disorders, and tocilizumab has been demonstrated to be highly efficacious in the treatment of rheumatoid arthritis, systemic juvenile idiopathic arthritis, and Castleman’s disease [40]. In terms of clinical implications, it has been reported that functional IL-6 receptor blocking is feasible and safe in epithelial ovarian cancer patients treated with carboplatin/(pegylated liposomal) doxorubicin plus tocilizumab [41]. Tocilizumab had a dramatic effect on cancer cachexia induced by IL-6–overexpressing lung cancer and prolonged survival without chemotherapy [42]. Another group also observed favorable responses to tocilizumab in terms of inflammation, nutritional status, anemia, and performance status in two patients with cancer-related cachexia [43]. It is suggested that serum IL-6 can be utilized as a surrogate marker for evaluating cachexia and prognosis of patients with chemotherapy-resistant metastatic lung cancer [44]. These reports imply that IL-6 and hepcidin may be key proteins involved in cancer-related anemia and suggest a rationale for targeting IL-6 in cancer-related symptoms. Regulation of IL-6 signaling might be one of the possible target pathways to treat IL-6-related disorders.

## Conclusions

We demonstrated here that blockade of the IL-6 signaling pathway by MR16-1 resulted in not only the suppression of elevated hepcidin levels leading to the improvement of iron-deficiency anemia status but also to amelioration of cancer symptoms, suggesting that suppression of hepcidin production by the blockade of IL-6 signaling may alleviate both anemia of cancer and cancer-related symptoms. Our data support the hypothesis that increased hepcidin levels via IL-6 signaling pathways play an etiologic role in cancer-related anemia by confining iron to cellular stores and not allowing exploitation of iron for hematopoiesis. This hypothesis provides a rationale for therapeutic targeting of IL-6 signaling pathways for managing anemia in cancer patients.

## Availability of data and materials

The dataset supporting the conclusions of this article is available in the LabArchives [http://www.labarchives.com/bmc] repository [https://mynotebook.labarchives.com/share/BCAN-D-15-00044/MjYuMHwxNzAzMTcvMjAvVHJlZU5vZGUvMzMyODM2NDczfDY2LjA=].

## Abbreviations

ACD: anemia of chronic disease
BFU-E: burst-forming units-erythroid
CFU-E: colony-forming units-erythroid
EDTA: ethylenediaminetetraacetic acid
EPO: erythropoietin
Hb: hemoglobin
HCT: hematocrit
IgG: immunoglobulin G
IL-1α: interleukin–α
IL-1β: interleukin-1β
IL-6: interleukin-6
LC/ESI–MS/MS: liquid chromatography/electrospray ionization tandem mass spectrometry
MCD: multicentric Castleman’s disease
MCH: mean corpuscular hemoglobin
MCV: mean corpuscular volume
MR16-1 group: MR16-1-treated LC-06-JCK–bearing mice
ND: not detected
NTB: non-tumor-bearing
nude mice: CAnN.Cg-Foxn1^nu^/CrlCrlj nu/nu mice
RBC: red blood cell
rhIL-6: recombinant human IL-6
SAA: serum amyloid A
STAT3: signal transducer and activator of transcription 3
TB group: IgG-treated LC-06-JCK–bearing control group
TNF-α: tumor necrosis factor-α
